# Bioactive Products From Plant-Endophytic Gram-Positive Bacteria

**DOI:** 10.3389/fmicb.2019.00463

**Published:** 2019-03-29

**Authors:** María J. Ek-Ramos, Ricardo Gomez-Flores, Alonso A. Orozco-Flores, Cristina Rodríguez-Padilla, Guadalupe González-Ochoa, Patricia Tamez-Guerra

**Affiliations:** ^1^Departamento de Microbiología e Inmunología, Facultad de Ciencias Biológicas, Universidad Autónoma de Nuevo León, San Nicolás de los Garza, Mexico; ^2^Departamento de Ciencias Químico Biológicas, División de Ciencias e Ingeniería, Universidad de Sonora, Navojoa, Mexico

**Keywords:** metabolites, amylases, chitinases, endoglucanases, esterases, proteases, plant hormones, toxins

## Abstract

Endophytes constitute plant-colonizing microorganisms in a mutualistic symbiosis relationship. They are found in most ecosystems reducing plant crops’ biotic and abiotic stressors by stimulating immune responses, excluding plant pathogens by niche competition, and participating in antioxidant activities and phenylpropanoid metabolism, whose activation produces plant defense, structural support, and survival molecules. In fact, metabolomic studies have demonstrated that endophyte genes associated to specific metabolites are involved in plant growth promotion (PGP) by stimulating plant hormones production such as auxins and gibberellins or as plant protective agents against microbial pathogens, cancer, and insect pests, but eco-friendly and eco-safe. A number of metabolites of Gram-positive endophytes isolated from agriculture, forest, mangrove, and medicinal plants, mainly related to the Firmicutes phyla, possess distinctive biocontrol and plant growth-promoting activities. In general, Actinobacteria and *Bacillus* endophytes produce aromatic compounds, lipopeptides, plant hormones, polysaccharides, and several enzymes linked to phenylpropanoid metabolism, thus representing high potential for PGP and crop management strategies. Furthermore, Actinobacteria have been shown to produce metabolites with antimicrobial and antitumor activities, useful in agriculture, medicine, and veterinary areas. The great endophytes diversity, their metabolites production, and their adaptation to stress conditions make them a suitable and unlimited source of novel metabolites, whose application could reduce agrochemicals usage in food and drugs production.

## Introduction to Endophytes

Endophytes are facultative or obligate symbiotic microorganisms, mainly bacterial and fungal species, that live in apparently healthy internal plant tissues, without causing disease (Schulz and Boyle, 2006). The most studied ones are bacterial and fungal species.

The purpose of this minireview is to highlight the importance of previously reported endophytic Gram-positive bacteria bioactive products. The International Union for Conservation of Nature and Natural Resources estimates that there are about 297,326 species of plants (Monocotyledons, Dicotyledons, Gymnosperms, Ferns and allies and Mosses), but only a few of them have been studied for their endophyte microbiota (Strobel and Daisy, 2003; Aitken, 2004). Endophytic microorganisms are known to influence plant physiology and development, among which, Gram-positive bacteria are important in such activities as bioremediation, biocontrol, plant growth, symbiotic-mutualistic, commensalistic, trophobiotic interactions, control of soil-borne pathogens, and support of host plant defense against environmental stress (Ryan et al., 2008). An endophytic community is complex and several factors may affect its structure, such as plant-microbe and microbe-microbe interactions and environmental conditions (Ryan et al., 2008). For bacterial endophytes diversity analysis, cultivation-based and culture-independent methods are used. In regard to cultivation studies, a great number of bacteria, mostly Proteobacteria, have been reported as endophytes, being the most frequent from Actinobacteria, Bacteroidetes, and Firmicutes phyla (Rosenblueth and Martínez-Romero, 2006).

**FIGURE 1:**
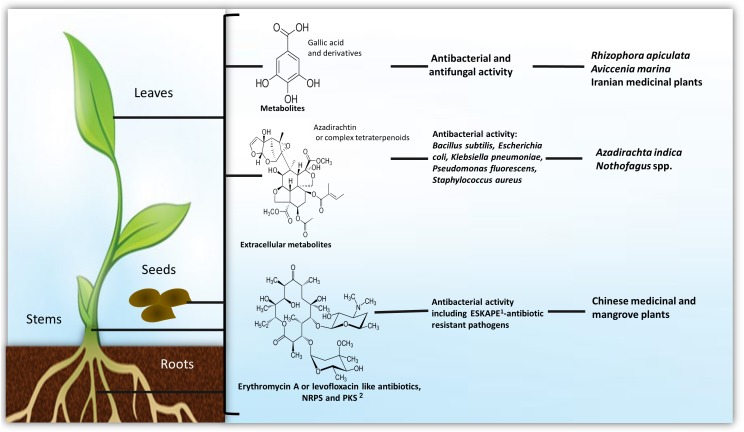
Production of metabolites by endophytes among plant tissues. ^1^The “ESKAPE” bacterial pathogens (*Acinetobacter baumannii, Enterobacter* spp., *Enterococcus faecium, Klebsiella pneumoniae, Pseudomonas aeruginosa*, and *Staphylococcus aureus*) are the leading nosocomial infectious agents throughout the world. ^2^Metabolites synthesized by non-ribosomal peptide synthetases (NRPS) or polyketide synthase (PKS). Detailed information on antimicrobial metabolites against animal/human pathogens are in Supplementary Table S1, against plant pathogens and insect pests in Supplementary Table S2, as plant growth stimulant in Supplementary Table S3 and as anti-cancer agent in Supplementary Table S4.

**FIGURE 2:**
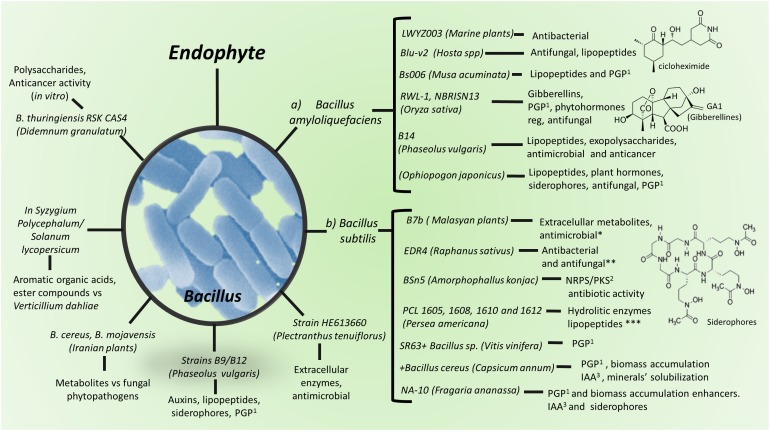
Production of metabolites by *Bacillus* spp. and strains as plant endophytes. (a) *Bacillus amyloliquefaciens*; (b) *Bacillus subtilis*. ^1^PGP, plant growth promoting. ^2^Metabolites synthesized by NRPS or PKS. ^3^IAA, indol acetic acid. Detailed information on antimicrobial metabolites against animal/human pathogens are in Supplementary Table S1, against plant pathogens and insect pests in Supplementary Table S2, as plant growth stimulant in Supplementary Table S3 and as anti-cancer agent in Supplementary Table S4.

**FIGURE 3:**
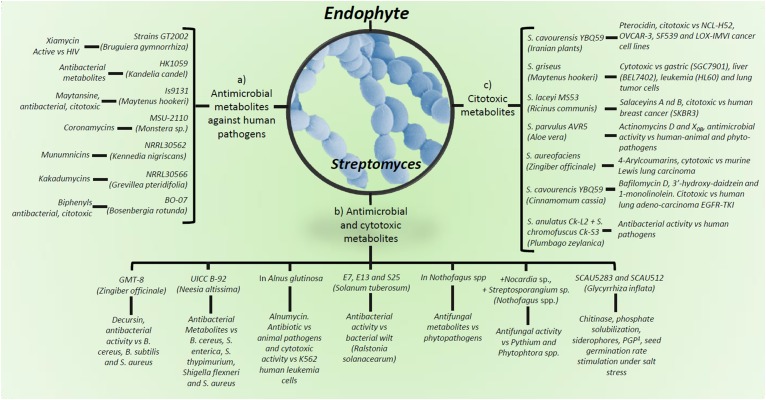
Production of metabolites by *Streptomyces* spp. and strains as plant endophytes. (a) Antimicrobial metabolites against human pathogens; (b) metabolites showing antimicrobial and cytotoxic activity; (c) cytotoxic metabolites against several tumor cell lines. ^1^PGP, plant growth promote. Detailed information on antimicrobial metabolites against animal/human pathogens are in Supplementary Table S1, against plant pathogens and insect pests in Supplementary Table S2, as plant growth stimulant in Supplementary Table S3 and as anti-cancer agent in Supplementary Table S4.

The most abundant metabolite producing Gram-positive bacteria endophytes found within diverse environments are *Bacillus* and *Streptomyces* species (Reinhold-Hurek and Hurek, 2011; Frank et al., 2017; Figure 1–3).

Endophytes are found in plants of most ecosystems and are of agricultural importance since they help to improve crops yields, by stimulating plants growth and immune response, excluding plant pathogens by niche competition, as well as actively participating in phenylpropanoid metabolism and antioxidant activities (Pandey et al., 2018). Among plant microbiota, endophytic bacteria can be found in most plant species and be recovered from roots, leaves, stems, and a few from flowers, fruits, and seeds (Lodewyckx et al., 2002); they have the potential to produce a variety of secondary metabolites with application in agriculture and pharmaceutical and industrial biotechnology (Lodewyckx et al., 2002; Strobel and Daisy, 2003; Ryan et al., 2008). Bacterial endophytes live within cell walls and xylem vessels intercellular regions and they may colonize seeds (Cankar et al., 2005; Johnston-Monje and Raizada, 2011), fruits (de Melo-Pereira et al., 2012), and flowers (Compant et al., 2011), among other tissues. It is known that endophytic bacteria are located in the apoplast (Koskimäki et al., 2015), and plant roots are proposed to be the entry point (Paungfoo-Lonhienne et al., 2010). It is also suggested that they are transmitted using an alternative vertical strategy due to their presence in flowers and seeds (Tamosiune et al., 2017). The potential explanation for their ubiquitous presence into plant tissues is the diversity of positive effects on plant growth and fitness they have shown, by stimulating the host phenylpropanoid pathway or by producing several linked-metabolites to the plants’ metabolism (Brader et al., 2014; Haidar et al., 2016; Lòpez-Fernàndez et al., 2016; Alaimo et al., 2018). Many reports indicate that bacterial endophytes help to provide nutrients as plant growth-promoters, and induce tolerance/resistance against biotic and abiotic stress conditions (Ryan et al., 2008).

In addition, several metabolites produced by microbial endophytes act as antimicrobial agents against human, animal, and plant pathogens. Whereas the antimicrobial effect against phytopathogens will have the positive effect on the host plant, the efficacies of endophyte metabolites may show a great clinical potential for medical and veterinary treatments. Indeed, nature-occurring antibiotics are low-molecular-weight products made by microbes that inhibit the growth or kill phytopathogens, bacteria, fungi, viruses, and protozoans, that cause human and animal diseases (Demain, 1981; Jakubiec-Krzesniak et al., 2018; Tripathi et al., 2018). Some important antibiotics producers have been recently found as endophytes in different plant species (Eljounaidi et al., 2016).

It is known that immunocompromised individuals (AIDS, cancer, and organ transplant patients) are at high risk for developing opportunistic microbial infections by *Aspergillus* sp., *Candida albicans, Clostridium difficile, Coccidioides immitis, Cryptococcus neoformans, Cryptosporidium, Mycobacterium avium* complex, *M. tuberculosis, Pneumocystis jirovecii, Pseudomonas aeruginosa, Salmonella* sp., *Staphylococcus aureus, Streptococcus pneumoniae*, and *S. pyogenes*, as well as parasitic infections caused by *Cryptosporidium* spp., *Encephalitozoon* spp., *Isospora belli, Leishmania* spp., *Plasmodium falciparum, Toxoplasma gondii*, and *Trypanosoma cruzi*. The urgent need for human diseases prevention and treatment, has promoted the discovery and development of novel and efficient therapeutic agents to which resistance has not been produced (Strobel and Daisy, 2003; Chinedum, 2005). For instance, drug resistance is a recognized phenomenon that disease-causing microbial agents develop against pharmaceutical therapy. Infectious diseases and cancer share similarities in the mechanisms of resistance to drugs, such as drug efflux, which is evolutionarily conserved (Housman et al., 2014). Therefore, in this review, the antibacterial, antifungal, antiviral, and antitumor activities of metabolites produced by specific Gram-positive bacteria endophytes are highlighted, as well as their potential use as plant growth promoters (PGP).

## Gram-Positive Endophytes Against Human/Animal Pathogens

Among Actinobacteria, Actinomycetes are Gram-positive, filamentous bacteria with great potential as biocontrol agents, which produce approximately two-thirds of natural antibiotics, where 75% derived from *Streptomyces* species (de Lima Procópio et al., 2012; Figure 1, 3). Actinomycetes, including the *Actinomyces, Actinoplanes, Amycolatopsis, Micromonospora, Saccharopolyspora*, and *Streptomyces* genera, are recognized as bioactive secondary metabolites producers, not only showing antimicrobial, but also insecticidal and antitumoral activities (Renu et al., 2008; Kekuda et al., 2010).

Lipopeptides are among the most important classes of secondary metabolites produced by endophytic bacteria, which are formed by cyclic or short linear peptides linked to a lipid tail or lipophilic molecules. Lipopeptides may show antimicrobial, cytotoxic and surfactant activities; they are synthesized by non-ribosomal peptide synthetases (NRPS), or polyketide synthase (PKS) and have great structural diversity based on a hydrophobic fatty acid acyl chain of 13 to 17 carbons, linked to a hydrophilic peptide of 7–25 aminoacids. Lipopeptides are important for both, their antibiotic activity and for inducing plant defense mechanisms (Stein, 2005; Raaijmakers et al., 2010). One bacterial strain may synthesize several polypeptide isoforms. *Bacillus* and *Paenibacillus*-related lipopeptides are the most studied ones (Villarreal-Delgado et al., 2018), whereas several *Bacillus amyloliquefaciens* strains have been recognized as higher lipopeptides producers (Figure 2A; Ongena and Jacques, 2008). In addition to lipopeptides, *B. subtilis* produces NRPS lantibiotics (lanthionine-containing antibiotics) (Stein, 2005); lipopeptides are responsible for biofilm and swarming development, whereas lantibiotics play as pheromones in quorum-sensing (Stein, 2005). *B. subtilis* also produces compounds such as polyketides, an aminosugar, and a phospholipid; polyketides include bacillomycin, fengycin, iturin, lichenysin, mycosubtilin, plipastatin, pumilacidin, and surfactin (Figure 2B); whereas *Paenibacillus polymyxa* synthesizes polymyxins (cyclic cationic lipopeptides) (Stein, 2005; Grady et al., 2016; Supplementary Tables S1–S4).

In addition to biologically active secondary metabolites, bacterial endophytes also produce important antimicrobial enzymes, mainly by Bacilli class members (Figure 2). In a study looking for highly producing enzymes endophytes, in the mangrove in Thailand, Khianngam et al. (2013) found that Gram-positive bacteria showed more hydrolytic activity compared with that of Gram-negative ones. Testing endophytes in hosts from the Rhizophoraceae family, results showed amylase, cellulase, and lipase activity by *B. infantis* and *B. granadenis*; and amylase, cellulase, lipase, lipolytic and proteinase activity by *B. safensis*. Similarly, cellulase, lipase, and proteinase activities by *Paenibacillus* sp. and *S. warneri* were detected in Acanthaceae family endophytes. Endophytes isolated from Brazilian mangrove plants showed high enzymatic activity; among these isolates, *Bacillus* sp. (MCR2.56) was reported to show particularly high amylase and esterasic activities; six *Bacillus* isolates (MCR2.51, MCA2.42, MCA2.51, MBR2.4, MBA2.33, and MBA2.4) high endocellulolytic activity, whereas the actinobacteria *Microbacterium* sp. (MCA2.54) and *Curtobacterium* sp. (MBR2.20) showed high endoglucanase and protease activity, respectively (Castro et al., 2014; Figure 2 and Supplementary Tables S1–S3).

Bioactive endophytic Streptomycetes can be isolated from plants worldwide (Castillo et al., 2007; Figure 3). Medicinal plants have been used for centuries as an alternative therapy for disease treatment. Interestingly, Chinese medicinal plants-endophytic actinomycetes were reported to have antibacterial activity against *E. coli* and *S. aureus* (Zhao et al., 2011; Figure 1). Recently, endophytic actinomycetes were reported in several Chinese mangrove plants to exhibit antibacterial activity against *Acinetobacter baumannii, Enterococcus faecali*s, *E. coli, Klebsiella pneumoniae, P. aeruginosa*, and *S. aureus*, some of which are resistant to the vancomycin, methicillin, and carbapenem antibiotics (Jiang et al., 2018; Figure 1, 3). Several metabolites from endophytic *Streptomyces* species have been characterized and associated with antibiotic activity, including kakadumycins, munumbicins, *p*-aminoacetophenonic acids, and xiamycins (Castillo et al., 2002, 2003; Guan et al., 2005); antimalarial (coronamycin, munumbicin D) (Castillo et al., 2002; Ezra et al., 2004); and antifungal (munumbicin D) (Shimizu et al., 2000; Ezra et al., 2004) activities (Figure 3 and Supplementary Table S1). Similarly, Iranian medicinal plants-endophytic actinomyctes exhibited antimicrobial activity against the pathogenic bacteria *B. cereus, B. subtilis, E. coli, Citrobacter freundii, K*. *pneumoniae, Proteus mirabilis, Shigella flexneri*, and *S. aureus* (Beiranvand et al., 2017; Figure 1, 3), whereas Malaysian plants-endophytic *B. subtilis* possessed antibacterial activity against *S. aureus*, methicillin resistant *S. aureus*, and *P. aeruginosa* (Fikri et al., 2018; Figure 2B). The African *Combretum molle*-endophytic *Bacillus* and *Lysinibacillus* species exhibited antibacterial activity against *B. cereus, E. coli, P. aeruginosa*, and *S. aureus* (Diale et al., 2018; Supplementary Table S1).

*Kennedia nigriscans*-endophytic *Streptomyces* sp. strain NRRL 30562 produces munumbicins A, B, C, and D, active against growth of *B. anthracis, E. faecalis*, vancomycin-resistant *E. faecalis*, multiple-drug-resistant (MDR) *M. tuberculosis, S. pneumoniae, S. aureus*, and methicillin-resistant *S. aureus*; furthermore, munumbicins have been shown to be more effective than chloroquine to kill the malaria-causing agent *P. falciparum* (Castillo et al., 2002, 2006; Christina et al., 2013; Figure 3A). Similarly, kakadumycins have antibacterial and antimalarial activities comparable with those of munumbicins (Waring and Wakelin, 1974; Katagiri et al., 1975; Castillo et al., 2003; Figure 3A and Supplementary Table S1).

*Streptomyces* sp. strain SUK06, isolated from the Malasian medicinal plant *Thottea grandiflora*, commonly used as an alternative mean to heal wounds and treat skin infections and fever, produces secondary antimicrobial metabolites against *B. cereus, B. subtilis, Plesiomonas shigelloides, P. aeruginosa*, and methicillin-resistant *S. aureus* (Ghadin et al., 2008). Similarly, metabolites and cell wall-degrading enzymes from *Panax ginseng*- and *Plectranthus tenuiflorus*-endophytic *Bacillus* sp., *Micrococcus* sp., and *P. polymyxa*, were reported to possess antibacterial activity against *E. coli, K. pneumoniae, P. mirabilis, Salmonella enterica* subsp. *enterica* serovar Typhi, *S. aureus*, and *S. agalactiae* (El-Deeb et al., 2013; Figure 2 and Supplementary Table S1).

Secondary metabolites spinosyn A and D, are produced by the soil actinomycete *Saccharopolyspora spinosa*, highly effective against lepidopteran and dipteran pests, among others, which commercial product named spinosad, has been commercialized for ∼250 countries and adopted in integrated pest management programs worldwide. Furthermore, there are reports of endophytic *Saccharopolyspora* species, although their potential as bioinsecticide has not yet been elucidated (Qin et al., 2011).

There are many medical and agricultural applications for the *Azadirachta indica* (known as neem) produced compounds (Figure 1). *Macrococcus caseolyticus* (ALS-1), a member of the Firmicutes, has been reported to produce free radical scavenging compounds. This strain was isolated from *Aloe vera* in an effort to cultivate bacterial endophytes that could be related to this plant curative and therapeutic uses (Akinsanya et al., 2015). In fact, 80% of the *A. vera* bacterial endophytes produced 1,1-diphenyl-2-picrylhydrazyl, showing over 75% scavenging properties. The *Raphanus sativus* (young radish) leaf and root endophytic *B. subtilis, Sphingobacterium siyangensis*, and *P. polymyxa* were shown to inhibit the growth of *B. cereus, E. coli, P. aeruginosa*, and *S. aureus*, in addition to *Salmonella, Shigella*, and *Listeria* species (Liu et al., 2010). *Zingiber officinale* roots-endophytic *Streptomyces* sp. was shown to possess antimicrobial activity against *B. cereus, B. subtilis*, and *S. aureus* (Taechowisan et al., 2013; Figure 3B and Supplementary Table S1).

## Gram-Positive Endophytes Against Plant Pathogens

Endophytes found in grapevine (*Vitis vinifera*) may represent one interesting example of a widely studied crop system. Metatranscriptoma analysis of vineyards prokaryotic microbiome confirmed that two out of three main bacterial phyla detected (Actinobacteria and Firmicutes) belonged to the Gram-positive group, thus reflecting bacterial metabolic assessments to become symbionts (either epiphytes or endophytes) and be distributed along plant tissues. This study demonstrated that the abundance and richness balance between beneficial microorganisms was critical for phytopathogens biocontrol and grapevine management, where the microbiota stability relied on environmental physicochemical conditions; being the soil type, geography and climate crucial factors to favor this crop. Moreover, detection of a specific strain reflected its ability to be established under the host microclimatic conditions, where *Bacillus* spp. were widely spread in flowers, leaves and grapes (Alaimo et al., 2018). In addition, Haidar et al. (2016) found that *B. pumilus* conferred systemic resistance against this pathogen, after studying the antagonistic bacteria modes of action for the phytopathogen *Phaeomoniella chlamydospora* biocontrol in grapevine.

The biological control of plant phytopathogens by endophytes was reported in late 50s where, a *Micromonospora* isolate from tomato showed antagonistic activity against *Fusarium oxysporum* f.sp. *lycopersici* (Manikprabhu and Li, 2016). As previously stated, the most abundant Gram-positive bacterial endophytes found within diverse environments are *Bacillus* and *Streptomyces* species (Reinhold-Hurek and Hurek, 2011; Frank et al., 2017), both exhibiting secondary metabolites showing antimicrobial activity also against plant pathogens. In fact, there have been proposed *Bacillus* spp. endophytes for crop management (Aloo et al., 2018; Figure 2). Similarly, *Streptomyces* spp. endophytes are widely reported as phytopathogens biocontrol agents (Figure 1, 3). For example, *K. nigriscans*-endophytic *Streptomyces* sp. strain NRRL 30562 were recently reported to produce antibiotics as munumbicins A, B, C, and D, active against plant pathogenic bacteria and fungi (Castillo et al., 2002). Leguminose plants-endophytic *Streptomyces caeruleatus* was reported to be effective against the soybean pathogen *X. campestris* pv. *glycine* (Mingma et al., 2014; Figure 3B); whereas metabolites from *A. indica*- and *Nothofagus* spp.-endophytic actinomyctes inhibited the plant pathogenic fungi *Mycosphaerella fijiensis, Sclerotinia sclerotiorum*, and *Rhizoctonia solani*, and *Pythium* and *Phytophthora* species (Castillo et al., 2007; Verma et al., 2009; Figure 1, 3B). Indeed, Castillo et al. (2007) reported *Streptomyces* spp. endophytic of *Nothofagus* spp. in southern Patagonia, where the same strain characterized as *Streptomyces seoulensis* (based on molecular sequenciation and biological activity) was isolated from two different plants (strains coded C2 and C4, respectively); their antifungal activity was then proposed to elucidate the native plants survival mechanisms against plant pathogens within that area (Figure 1, 3B). Similarly, metabolites produced by roots’ endophytic actinomycetes previously described (Matsumoto and Takahashi, 2017), inhibited *Kocuria rhizophila* strain KB-212, *Mucor racemosus* strain KF-223, and *Xanthomonas campestris* pv. *oryzae* strain KB-88 growth (Figure 1 and Supplementary Table S2).

*Zea mays* seeds-endophytic *B. amyloliquefaciens* and *B. subtilis* were observed to inhibit *F. moniliforme* fungus growth by producing lipopeptides (Gond et al., 2015); similarly, *Bruguiera gymnorrhiza* (L.) Lam-endophytic *B. amyloliquefaciens* was shown to be antagonistic to various bacterial (*Ralstonia solanacearum, P. syringae*, and *X. campestris*), and fungal (*Colletotrichum musae, F. oxysporum, Phytophthora capsici*, and *R. solani*) pathogens of plants and to be effective in the biocontrol of *Capsicum* bacterial wilt in pot and field trials (Hu et al., 2010).

*Oryza sativa*-endophytic *B. cereus* and *B. mojavensis* were observed to exhibit antimicrobial activity against the fungal rice pathogens *F. fujikuroi, F. proliferum, F. verticillioides, Magnaporthe grisea*, and *M. salvinii* (Etesami and Alikhani, 2017; Supplementary Table S2).

Young radish-endophytic *B. subtilis, Brachybacterium*, and *P. polymyxa* were reported to possess antifungal activity against *F. oxysporum, Pythium ultimum, Phytophthora capsici*, and *R. solani* (Seo et al., 2010). An antifungal protein from the wheat-endophytic *B. subtilis* strain EDR4 inhibited *B. cinerea, F. graminearum, F. oxysporum* f.sp. *vasinfectum, G. graminis* var. *tritici, Macrophoma kuwatsukai*, and *R. cerealis* growth (Liu et al., 2010; Supplementary Table S2).

## Metabolites From Gram-Positive Endophytes as Plant Growth-Promoters

Endophytic bacteria use to protect crops from microbial diseases is relevant, for their potential to promote host growth and antimicrobial activity (Safiyazov et al., 1995; Berg et al., 2005). Plant growth promotion (PGP) and, in most cases, abiotic stress tolerance and disease protection properties induction, are associated with endophytic bacteria potential to produce different compounds. Plants acclimate to environmental stresses by altering their physiology to be able to overcome stress factors such as dehydration, mechanical injury, nutrient deficiency, high solar radiation, or biotic/abiotic factors. It has been observed that plant inoculation with endophytic bacteria leads to accumulation of “protective” compounds, such as proline, carbohydrates, and antioxidants, in addition to antibiotics and fungal cell-wall lytic enzymes, which can inhibit growth of plant pathogens (Brader et al., 2014) or prime plant response to pathogens by induced systemic resistance (ISR) mechanisms (Pieterse et al., 2014).

Bacterial endophytes PGP potential is explained through several proposed mechanisms. Several of which help to increase accessibility to nutrients, e.g., nitrogen and phosphorus or metals, or produce metabolites that could regulate plant growth, development and defense responses, such as the well-known phytohormones abscisic acid, auxins, brassinosteroids, cytokinins, ethylene, gibberellins, jasmonates, and strigolactones (Reinhold-Hurek and Hurek, 2011; Brader et al., 2014; Santoyo et al., 2016; Shahzad et al., 2016; Figure 1–3).

Some examples of Gram-positive PGP bacterial endophytes are *B. pumilus* strain E2S2, whose treatment increased roots and shoots length and fresh and dry biomass, as compared with untreated sorghum plants, and helped to augment cadmium uptake (Luo et al., 2012). *B. amyloliquefaciens* strain NBRI-SN13 (SN13) isolated from an alkaline soil of Banthara, Lucknow, India, showed several PGP attributes and to induce solubilization of tricalcium phosphate more efficiently, when inoculated as endophyte (Nautiyal et al., 2013; Figure 2A). Plants treated with *B. atrophaeus* strain EY6, *B. sphaericus* GC subgroup B EY30, *B. subtilis* strain EY2, *S. kloosii* strain EY37 and *K. erythromyxa* strain EY43 as endophytes, have been shown to increase strawberry fruit growth and yield (Karlidag et al., 2011; Figure 2).

Interestingly, *C. botulinum* strain 2301 has been shown to have a significant PGP effect on clover in field experiments (Zeiller et al., 2015); whereas *Exiguobacterium acetylicum* 1P strain MTCC 8707, a cold tolerant bacterial strain from the Uttarakhand Himalayas, promotes wheat seedlings growth (Selvakumar et al., 2010; Supplementary Table S3).

*Brevibacillus* brevis strain SVC(II)14 exerted beneficial PGP on cotton crop (Nehra et al., 2016). *Bacillus* spp. strains CPMO6 and BM17, actinobacteria isolates ACT01 and ACT07, and lactic acid bacteria strain BL06 induce phosphate solubilization more efficiently when present as endophytes in citrus (Giassi et al., 2016).

In recent years, it has also been demonstrated that the entomopathogenic bacteria *B. thuringiensis* can have PGP attributes. Armada et al. (2016) tested an autochthonous isolate in interaction with native arbuscular mycorrhizal fungi (single or mixture) and found stimulating plant growth, nutrition and drought tolerance responses.

Siderophores production by endophytes improves plant growth by binding to available iron, competing for this element with phytopathogens and protecting the host plant from their infection (Figure 2, 3; Sabaté et al., 2018). *B. subtilis* strain B26 has been shown to induce drought tolerance in *Brachypodium distachyon* grass. This was correlated to augmentation of starch, fructose, glucose and total soluble carbohydrates content. However, increase of raffinose-related family carbohydrates (well-known stress response metabolites) was not observed in control and treated plants (Gagné-Bourque et al., 2015). A proline accumulation stimulating effect by endophytic strains of the actinobacteria *Arthrobacter* sp. and the Firmicutes *Bacillus* spp. were reported in pepper (*Capsicum annuum* L., Solanales: Solanaceae) plants *in vitro*, where their synthesis was related to osmotic stress responses (Sziderics et al., 2007). In addition, plants inoculated with bacterial endophytes, could tolerate abiotic stresses by increasing enzymatic activity. *B. cereus* strain CSR-B-1, *B. marisflavi* strain CSR-G-4, *B. pumilus* strain CSR-B-2, *B. saffensis* strain CSR-G-5, *B. subtilis* strain CSR-G-1, and *B. thuringiensis* strain CSRB-3, induced increment of superoxide dismutase, phenylalanine lyase, catalase, and peroxidase enzymes activity in gladiolus plants under sodium high concentration conditions (Damodaran et al., 2014; Supplementary Table S3).

Tolerance to low temperatures and growth promotion by endophytic activity has been reported as well. Verma et al. (2015), found *Bacillus* and *Bacillus* derived genera as wheat (*Triticum aestivum*) endophytes from the northern hills zone of India, among others. Phosphate and potassium are major essential macronutrients, but soluble phosphate and potassium concentrations in soil for plant intake are usually very low. Plants need zinc at low concentration since it is toxic at high concentration, thus zinc solubilization by endophytes dosifies the plant intake amount in response to plant and microbial nutritional requests. The most efficient phosphate solubilizing Gram-positive bacteria (PSB) belong to the genera *Bacillus*. Besides, it has been reported that *B. amyloliquefasciens, B. megaterium*, and *Bacillus* sp., exhibit phosphorus, potassium, and zinc solubilization (Figure 2; Verma et al., 2015).

## Anticancer Activity of Gram-Positive Endophytic Bacteria

Cancer prevails as one of the leading causes of death worldwide, in spite of therapy advances (Global Cancer Observatory [GLOBOCAN], 2008; Chen et al., 2013). Conventional chemotherapy and radiotherapy have important disadvantages including drug resistance and serious side effects, which has prompted the search for new antitumor agents with high therapeutic efficacy and marginal or null detrimental effects.

Many endophytic actinomycete compounds were isolated and have found application not only as antimicrobial agents but also as cytotoxic agents against tumor cells (Figure 1, 3). Some members of the Gram-positive bacteria group have been recently found as endophytes in different plant species (Eljounaidi et al., 2016). Endophyte extracts have demonstrated to be a better choice versus chemotherapy agents due to their antitumor activity efficacy and lower side-effects, since they are less toxic to normal cells and more effective against several drug resistant microorganisms. As a consequence, the natural endophyte-derived metabolites have attracted peculiar attention with the purpose of being human cancer-chemopreventive compounds and anticancer chemotherapeutic drugs (Cardoso-Filho, 2018; Figure 3B,C). Endophytic Gram-positive bacterial natural products have emerged as one of the most reliable alternative treatment sources (Gutierrez et al., 2012; Chen et al., 2013), including antitumor agents such as anthracyclines, anthraquinones, aureolic acids, β-glucans, carzinophilin, coumarins, enediynes, flavonoids, glycopeptides, macrotetrolides, mitomycins, naphthoquinones, polysaccharides, and quinoxalines (Waring and Wakelin, 1974; Igarashi et al., 2007; Taechowisan et al., 2007; Chen et al., 2013; Cardoso-Filho, 2018; Figure 3B,C and Supplementary Table S4).

Actinomycetes, including the genera *Actinomyces, Actinoplanes, Amycolatopsis, Micromonospora, Saccharopo-lyspora*, and *Streptomyces* are recognized as producers of bioactive metabolites with not only antimicrobial, but also antitumor potential (Renu et al., 2008; Kekuda et al., 2010; Figure 3C and Supplementary Table S4). In this concern, *Ophiopogon japonicus*-endophytic *B. amyloliquefaciens* sp. exopolysaccharides were reported to possess antitumor activity against the human gastric carcinoma cell lines MC-4 and SGC-7901 (Chen et al., 2013; Figure 2A). Furthermore, *Maytenus hookeri*-maytansine-producing endophytic *Streptomyces* sp. strain Is9131 inhibited human SGC7901 gastric, HL60 leukemia, BEL7402 liver, and A-549 lung tumor cell lines growth (Lu and Shen, 2003; Zhao et al., 2005; Figure 3B and Supplementary Table S4). *Alnus glutinosa*-endophytic *Streptomyces* alnumycin was reported to inhibit the growth of K562 human leukemia cells (Bieber et al., 1998; Figure 3C), whereas *Ricinus communis*-endophytic salaceyins-producing *Streptomyces laceyi* strain MS53 was observed to be cytotoxic against the human breast cancer cell line SKBR3 (Kim et al., 2006; Figure 3B). In addition, herbaceous and arbor plants-pterocidin-producing endophytic *Streptomyces hygroscopicus* strain TP-A0451 was reported to inhibit human cancer cell lines NCI-H522, OVCAR-3, SF539, and LOX-IMVI growth (Igarashi et al., 2006; Qin et al., 2011), and *Z. officinale* 4-arylcoumarins-producing-endophytic *Streptomyces aureofaciens* strain CMUAc130 was shown to be cytotoxic against murine Lewis lung carcinoma (Taechowisan et al., 2007; Qin et al., 2011; Figure 3C and Supplementary Table S4).

Many plant growth promoter compounds have shown cytotoxicity against tumor cells. Nodules of *Lupinus angustifolius*-endophytic anthraquinones-producing *Micromo-nospora* sp. actinomycete significantly inhibited invasion of murine colon 26-L5 carcinoma cells (Igarashi et al., 2007; Qin et al., 2011). Recently, Taechowisan et al. (2017) reported anticancer activity of *Boesenbergia rotunda*-endophytic biphenyls-producing *Streptomyces* sp. strain BO-07 against human HepG2 and Huh7 liver, and HeLa cervical tumor cell lines (Figure 3B). Furthermore, *Cinnamomum cassia*-endophytic *Streptomyces cavourensis* strain YBQ59 was shown to inhibit human lung adenocarcinoma EGFR-TKI-resistant cells A549 and H1299 growth (Vu et al., 2018; Figure 3C and Supplementary Table S4).

## Conclusion and Perspectives

Since the first reports of the industrial potential use of secondary metabolites produced by endophytes, there is more evidence that endophytic Gram-positive bacteria are one of the most important sources of novel compounds that have proven potential for either agriculture, medical and/or pharmaceutical application, thanks to their PGP, antimicrobial and anticancer activities. Indeed, endophytic Gram-positive bacteria help plants to better survive under biotic and abiotic stress conditions. It is not random that many endophytic Gram-positive have been isolated from medicinal plants from all over the world. In fact, many reports of endophytic Gram-positive bacteria showing such activities have been isolated from mangrove and under extreme environment conditions growing plants, like crops from salty soils and crops and trees from cold areas, where phylogenic analysis of native strains demonstrate they contain genes to produce different metabolites that, all together, are helping the host plant, not just to survive, but also to improve the plant adaptation to these extreme environmental conditions. In general, *Bacillus* class endophytes have been reported to produce aromatic compounds, lipopeptides, plant hormones, polysaccharides, and several enzymes, thus representing higher potential in agriculture for PGP and crop management strategies (Villarreal-Delgado et al., 2018). Similarly, Actinobacteria class endophytes are being found to produce antimicrobial- and antitumor-like activity metabolites, thus representing high potential for agriculture, medical, and veterinary application. In this minireview, we presented sources and specific isolated strains information, with the aim to provide current research highlights and perspectives for their future applications. These fascinating microorganisms are diverse and greatly adaptable to extreme stress conditions, making them excellent novel metabolites sources, whose application would be important for environmentally friendly and sustainable food and drugs production.

## Author Contributions

All authors listed have made a substantial, direct and intellectual contribution to the work, and approved it for publication.

## Conflict of Interest Statement

The authors declare that the research was conducted in the absence of any commercial or financial relationships that could be construed as a potential conflict of interest.
