# Supplementary material for: Bioactive Products From Plant-Endophytic Gram-Positive Bacteria
Source: Front Microbiol. 2019 Mar 29;10:463. doi: 10.3389/fmicb.2019.00463 (PMC6449470; doi:10.3389/fmicb.2019.00463)
Supplement: Supplementary file 1 [file Data_Sheet_1.pdf]

Supplementary material

Supplemental Table S1. Gram positive endophyte-derived antimicrobial metabolites against animal/human pathogens

Gram positive endophyte	Plant source	Chemical agent	Reported Activity against:	Reference
Actinomycetes	*Avicennia marina* (Forssk.) Vierh. (Lamiales: Acanthaceae); *Rhizophora apiculata* Blume (Malpighiales: Rhizophoraceae)	Metabolites	Antibacterial	Gayathri and Muralikrishnan, 2013
Actinomycetes	*Azadirachta indica* A. Juss (Sapindales: Meliaceae) *Nothofagus* spp. Blume (Fagales: Nothofagaceae)	Extracellular metabolites	*Bacillus subtilis, Escherichia coli, Klebsiella pneumoniae, Pseudomonas fluorescens* and *Staphylococcus aureus*	Verma et al., 2009
Actinomycetes	Chinese mangrove plants	Erythromycin- and levofloxacin-like antibiotics	Antibacterial including ESKAPE[1]- antibiotic resistant pathogens	Jiang et al., 2018
Actinomycetes	Chinese medicinal plants	NRPS and PKS[2]	Antibacterial<br>Antifungal	Zhao et al., 2011
Actinomycetes	Iranian medicinal plants	Metabolites	Antibacterial	Beiranvand et al., 2017
Bacillus spp.	*Solanum lycopersicum* L. (Solanales: Solanaceae)	Extracellular metabolites	*Bacteria: Bacillus subtilis, E. coli,* and *S. aureus*<br>*Fungus: Aspergillus niger*	Tian et al., 2017
Bacillus spp.	*Syzygium polycephalum* Miq. (Merr & Perry) (Myrtales: Myrtatceae)	Extracellular metabolites	*B. cereus, Klebsiella pneumoniae*, and methicillin-resistant *S. aureus*	Indrawati et al., 2018
Bacillus sp. strain HE613660	*Plectranthus tenuiflorus* (Vatke)	Extracellular enzymes (amylase, cellulose, esterase,	*Bacteria: E. coli, K. pneumoniae, Proteus mirabilis, S. aureus,*	El-Deeb et al., 2013

	(Lamiaales: Lamiaceae)	lipase, pectinase, protease, and xylanase)	*Streptococcus agalactiae*; *Fungus: Candida albicans*	
Bacillus spp. and *Lysinibacillus*	*Combretum molle* R. Br. Ex G. Don (Myrtales: Combretaceae)	Antibacterial metabolites	*Bacillus cereus, E. coli, P. aeruginosa* and *S. aureus*	Diale et al., 2018
Bacillus amyloliquefaciens	*Bruguiera gymnorrhiza* (L.) Savigny (Malpighiales: Rhizophoraceae)	Metabolites	Antibacterial, Antifungal	Hu et al., 2010
B. amyloliquefaciens	*Triticum aestivum* L. (Poales: Poaceae)	Protein	Antifungal	Liu et al., 2010
B. amyloliquefaciens LWYZ003	Marine plants	Cicloheximide	Antibacterial	Liu et al., 2018
B. amyloliquefaciens, *Bacillus subtilis*	*Zea mays* L. (Poales: Poaceae)	Lipopetides, Metabolites	Antifungal	Gond et al., 2015
Bacillus cereus and *Bacillus mojavensis*	Iranian plants	Metabolites	*Fusarium fujikuroi*, *Fusarium proliferum*, *Fusarium verticillioides*, *Magnaporthe grisea*, and *Magnaporthe salvinii*	Etesami and Alikhani, 2017
B. subtilis strain B7b	Malaysian (Pulau Langkawi) plants	Extracellular metabolites	Bacteria: *Pseudomonas aeruginosa* and methicillin-resistant *S. aureus* Fungi: *Colletotrichum gloeosporioides* and *Fusarium oxysporum*	Fikri et al., 2018
B. subtilis strain EDR4	*Raphanus sativus* L. (Brassicales: Brassicaceae)	Metabolites	Antibacterial, Antifungal	Seo et al., 2010
Brevibacterium sp. strain YXT131	*Camellia sinensis* var. *assamica L.* Kuntze (Ericales: Theaceae)	NRPS and PKS[2]	*Escherichia coli*, *Shigella flexneri*, and *Staphylococcus epidermidis*	Wei et al., 2018
Paenibacillus polymyxa	*Triticum aestivum* L. (Poales: Poaceae)	Fusaricidin A–D	Antifungal	Beck *et al.*, 2003
Streptomyces sp.	*Bruguiera*	Xiamycin	Human immune-	Ding et al.,

strain GT2002/1503	*gymnorrhiza L. Lam.* (Malpighiales: Rhizophoraceae)		deficiency virus (HIV)	2010
Streptomyces sp. strain HKI0595	*Kandelia candel* L. (Malpighiales: Rhizophoraceae)	Indosespene, sespenine, and xiamycin B, multicyclic indolo-sesquiterpenes	*Bacillus subtilis*, vancomycin-resistant *Enterococcus faecalis*, *Mycobacterium vaccae*, *P. aeruginosa*, *S. aureus*, and methicillin-resistant *S. aureus*	Ding et al., 2011
Streptomyces sp. strain Is9131	*Maytenus hookeri* Molina (Celastrales: Celastraceae)	Maytansine (dimeric dinactin macrolid)	*M. tuberculosis* and *S. aureus*	Zhao et al., 2005
Streptomyces sp. strain MSU-2110	*Monstera* sp. Adans (Arismatales: Araceae)	Coronamycins (peptide)	Antibiotics	Ezra *et al.*, 2004
Streptomyces sp. strain NRRL30562	*Kennedia nigriscans* Lindl. (Fabales: Fabaceae)	Munumbicins, munumbicin D	Bacteria as: *Bacillus anthracis*, MDR[3]-*Mycobacterium tuberculosis* and *S. aureus*.<br>Protozoa: *Plasmodium falciparum*	Castillo et al., 2002
Streptomyces sp. strain NRRL30566	*Grevillea pteridifolia Knight* (Proteales: Proteaceae)	Kakadumycins	Antibiotic	Castillo *et al.*, 2003
Streptomyces sp. strain BO-07	*Boesenbergia rotunda* (L.) Mansf (Zingiberales: Zingiberaceae)	Biphenyls	*B. cereus*, *B. subtilis* and *S. aureus*	Taechowisan et al., 2017
Streptomyces sp. strain GMT-8	*Zingiber officinale* Rosc. (Zingiberales: Zingiberaceae)	Decursin	*B. cereus*, *B. subtilis* and *S. aureus*	Taechowisan et al., 2013
Streptomyces sp. strain UICC B-92	*Neesia altissima* (Blume) Blume (Malvales: Malvaceae)	Antibacterial metabolites	*B. cereus*, *S. enterica* servoar. Typhimurium, *Shigella flexneri*, and *S. aureus*	Pratiwi et al., 2018
Streptomyces sp.	*Alnus glutinosa* Gaertn. (Fagales: Betulaceae)	Alnumycin (exfoliamycin- and naphthapyranomycin - like activity)	*Arthrobacter crystallopoites*, *Mirococcus luteus*, and *Rhodococcus* sp.	Bieber et al., 1998
Streptomyces	*Plumbago*	Antibacterial	*B. cereus*, *B. subtilis*, *E.*	Chandrakar

anulatus strain Ck-L2; *Streptomyces chromofuscus* strain Ck-S3	*zeylanica* L.	metabolites	*coli*, *K. pneumoniae*, *P. vulgaris*, *P. aeruginosa*, *S. aureus*, and *S. epidermidis*	and Gupta, 2017
Streptomyces griseus	*Kandelia candel*	*p*-Amino-acetophenonic acids	Antimicrobial	Guan *et al.*, 2005
Streptomyces parvulus strain Av-R5	*Aloe vera* L. Burm. (Asparagales: Xanthorrhoeaceae)	Actinomycins D and $X_{0\beta}$	*Bacteria: K. pneumoniae*, *Proteus vulgaris*, *P. aeruginosa*, MRD[3]- *S. aureus*, *S. epidermidis*, *Fungi: Aspergillus niger*, *Candida albicans*	Chandrakar and Gupta, 2018

[1] The "ESKAPE" bacterial pathogens (*Acinetobacter baumannii*, *Enterobacter* spp., *Enterococcus faecium*, *Klebsiella pneumoniae*, *Pseudomonas aeruginosa*, and *Staphylococcus aureus*) are the leading nosocomial infectious agents throughout the world.

[2]Synthesized by nonribosomal peptide synthetases (NRPS) or polyketide synthase (PKS)

[3]Multidrug-resistent

**Supplemental Table S2. Gram positive endophyte-derived antimicrobial metabolites against plant pathogens and insect pests**				
**Gram positive endophyte**	**Plant source**	**Chemical agent**	**Reported activity against**	**Reference**
Actinokineospora SCAU5231 *Streptomyces* spp. strains SCAU5283 and SCAU5215	*Glycyrrhiza inflata* Batalin (Fabales: Fabaceae)	Antimicrobial metabolites	Bacteria as *E. coli* and *S. aureus*. Fungi as *Alternaria alternate*, *Curvularia lunata*, *Fusarium graminearum*, *F. oxysporum*, and *Mycogone perniciosa*	Zhao et al., 2018
Arthrobacter spp., *Bacillus* spp., *Exiguobacterium antarcticum*, *Kocuria kristinae*	*Triticum aestivum* L. (Poales: Poaceae)	Antifungal	*Fusarium graminearum*, *Macrophomina phaseolina* and *Rhizoctonia solani*	Verma et al., 2015
B. amyloliquefaciens strain Blu-v2	*Hosta* spp., Tratt (Asparagales: Asparagaceae)	Lipopeptides, metabolites	Antifungal, caterpillar-feeding deterrant	Li et al., 2015
B. amyloliquefaciens	*Musa acuminate* Colla (Zingiberales: Musaceae)	Phenolic acids. Antimicrobial and PGP[1] metabolites	Phytopathogens control and PGP	Yuan et al., 2018; Gamez et al., 2019
B. amyloliquefaciens strain B14, and *Bacillus* sp. strains B19 and P12	*Phaseolus vulgaris* L. (Fabales: Fabaceae)	Lipopeptides	White mold (*Sclerotinia sclerotiorum*)	Sabaté et al. 2018
Bacillus atrophaeus strain XEGI50	*Glycyrrhiza uralensis* Fisch. (Fabales: Fabaceae)	Aromatic organic acids, and ester compounds	Wilt fungus *Verticillium dahliae*	Mohamad et al., 2018
Bacillus subtilis BSn5	*Amorphophallus konjac* K. Koch (Arismatales: Araceae)	NRPS/PKS[1] antibiotics and lantibiotics	Soft rot disease *Erwinia carotovora* subsp. *carotovora*	Deng et al., 2011
B. subtilis (four strains)	*Persea americana* Mill. (Laurales:	Hydrolytic enzymes; surfactin, fengycin, and iturin A	*Fusarium oxysporum* f. sp. *radices - lycopersici* and	Cazorla et al., 2007

	Lauraceae)	(lipopeptides)	*Rosellinia necatrix*	
Bacillus velezensis strain CC09	*Cinnamomum camphora* (Laurales: Lauraceae)	NRPS and trans-acyl transferase PKS[1]	Wheat powdery mildew disease (*Blumeria graminis* f. sp. *tritici*)	Cai et al., 2017
Curtobacterium sp.	*Vitis vinifera* L. Duchesne (Vitales: Vitaceae)	Antibacterial metabolites	Grape crown gall disease (*Agrobacterium vitis*)	Ferrigo et al., 2017
Micromonospora chokoriensis strain SAUK6030, *Nonomuraea roseola* strain SAUK6015	Chinese medicinal plants	NRPS and PKS[1]	*Curvularia lunata*, responsible for seed germination failure and seedling blight in monocotyledon crops	Zhao et al., 2011
Nonomuraea roseola strain SAUK6015	Chinese medicinal plants	NRPS and PKS[1]	Maize leaf blight *(Exerohilum turcicum)*	Zhao et al., 2011
Streptomyces caeruleatus strain MR365	*Glycine max* (L.) Merr. (Fabales: Fabaceae)	Antibacterial metabolites	Bacterial leaf pustule *(Xanthomonas campestris* pv. *glycines)*	Mingma et al., 2014
Streptomyces spp., strains E7, E13 and S25	*Solanum tuberosum* L. (Solanales: Solanaceae)	Antibacterial metabolites	*Bacterial wilt* *(Ralstonia solanacearum)*	Rado et al., 2015
Streptomyces spp.	*Nothofagus spp. Blume (Fagales: Nothofagaceae)*	Antifungal metabolites	*Mycosphaerella fijiensis, Pythium ultimum, Phytophthora erythroseptica*, *Rhizoctonia solani* and *Sclerotinia sclerotiorum*	Castillo et al., 2007
Streptomyces sp., *Nocardia* sp., and *Streptosporangium* sp.	*Azadirachta indica Nothofagus* spp.	Extracellular metabolites	*Pythium* and *Phytophthora* sp.	Verma et al., 2009
Streptosporangium oxazolinicum strain K07-0450T	*Orchid* sp. Juss (Asparagales: Orchidaceae)	Spoxazomicins A-C	Anti-trypanosomal (*Trypanosoma brucei brucei*)	Inahashi et al., 2011a, b

[1]Synthesized by nonribosomal peptide synthetases (NRPS) or polyketide synthase (PKS)

**Supplemental Table S3. Gram positive endophyte-derived metabolites as plant growth promoters (PGP)**				
**Gram positive endophyte**	**Plant source**	**Chemical agent/ enzymatic activity**	**Reported Activity**	**Reference**
Arthrobacter spp., *Bacillus* spp., *Exiguobacterium antarcticum*, *Kocuria kristinae*	*Triticum aestivum* L. (Poales: Poaceae)	Several PGP[1] metabolites	PGP[1]	Verma et al., 2015
Bacillus spp.	*S. lycopersicum*	Ammonia, metabolites	Dinitrogen fixation	Tian et al., 2017
Bacillus spp., *B. subtilis* strain SR63	*Vitis vinifera* L. Duchesne (Vitales: Vitaceae)	Metabolites	PGP[1]	Baldan et al., 2014; Ferrigo et al., 2017
Bacillus spp., *Brevibacillus* sp., *Lysin ibacillus* sp., *Paenibacillus* sp., *Staphylococcus* sp.	*S. lycopersicum*	Extracelluar metabolites	PGP[1] and biomass improvement	Xia et al., 2015
Bacillus amyloliquefaciens strain RWL-1	*Oryza sativa* L. (Poales: Poaceae)	Gibberellins (hormone)	PGP[1], hormone regulation	Shahzad et al., 2016
B. amyloliquefaciens strain B14, and *Bacillus* sp. strains B19 and P12	*Phaseolus vulgaris*	Auxines, siderophores	PGP[1], seed germination	Sabaté et al., 2018
Bacillus cereus and *Bacillus mojavensis*	Iranian plants	Metabolites	PGP[1]	Etesami and Alikhani, 2017
B. amyloliquefaciens B. cereus and *Bacillus subtilis*	*Capsicum annuum* L. (Solanales: Solanaceae)	Indol acetic acid and phosphorus, potassium and zinc solubilisation	Anthracnose control, PGP[1] and biomass improvement	Peña-Yam et al., 2016; Gowtham et al., 2018
B. subtilis strain EA-CB0575	*M. acuminate* and *S. lycopersicum*	Metabolites	PGP[1]	Posada et al., 2018
B. subtilis strain NA-108	*Fragaria* x *ananassa* Duchesne (Rosales: Rosaceae)	Indol acetic acid and siderophores	PGP[1] and biomass improvement	de Melo-Pereira et al., 2012
Brevibacterium	*Oryza sativa*	Antioxidant and H^+	Salt tolerance	Chatterjee et al.,

linens strain RS16		ATPase activity regulation		2018
Streptomyces sp.	*Beta vulgaris* L. (Caryophyllales: Amaranthaceae); *V. vinifera*	Several PGP metabolites[1]	PGP[1]	Baldan et al., 2014; Tsurumaru et al., 2015
Streptomyces spp. strains SCAU5283 and SCAU5215	*Glycyrrhiza inflata*	Chitinases, phospate solubilization, siderophores	PGP[1], seed germination rate stimulation under salt stress	Zhao et al., 2018

[1]Plant growth promoters (PGP) such as: ammonia, hydrogen cyanide, indole-3-acetic acid and siderophore production, phosphorus, potassium and zinc solubilisation; 1-aminocyclopropane-1-carboxylate deaminase activity

Supplemental Table S4. Gram positive endophyte-derived antimicrobial metabolites against cancer cells

Gram positive endophyte	Plant source	Chemical agent	Reported Activity	Reference
B. amyloliquefaciens	*Ophiopogon japonicus* (Thunb.) Ker Gawl. (Asparagales: Nolidoideae)	Exopoly-saccharides	Human gastric carcinoma MC-4 and SGC-7901 cell lines	Chen et al., 2013
Bacillus thuringiensis strain RSK CAS4	*Didemnum granulatum* Tokioka (marine invertebrate)	Polysaccharides	Human A549 lung and HEp-2 liver cancer cell lines	Ramamoorthy et al., 2018
Brevibacterium sp. strain YXT131	*C. sinensis* var. *assamica*	Extracellular metabolites	Decrease of interleukin 12 and 23 pro-inflammatory cytokines, and tumor necrosis factor-α	Wei et al., 2018
Micromonospora lupini	*Lupinus angustifolius* L. (Fabales: Fabaceae)	Lupinacidins A and B (anthraquinones)	Murine colon 26-L5 carcinoma cells	Igarashi et al., 2007
Streptomyces sp.	*Alnus glutinosa*	Alnumycin (exfoliamycin- and naphthapyranomycin-like activity)	K562 human leukemia cells	Bieber et al., 1998
Streptomyces sp.	*Maytenus hookeri* Loes. (Celastrales: Celastraceae)	Maytansine	A-549 lung tumor cell	Lu and Shen, 2003
Streptomyces sp. strain Is9131	*Maytenus hookeri*	Maytansine (dimeric dinactin macrolid)	Gastric (SGC7901), liver (BEL7402), luekemia (HL60), and lung (A549) tumor cells	Zhao et al., 2005
Streptomyces sp. strain BO-07	*Boesenbergia rotunda*	Biphenyls	HeLa, HepG2 and Huh7 cancer cell lines	Taechowisan et al., 2017
Streptomyces aureofaciens strain CMUAc130	*Zingiber officinale*	4-Arylcoumarins	Murine Lewis lung carcinoma	Taechowisan et al., 2007
Streptomyces	*Cinnamomum*	Bafilomycin D, 3′-	Human lung	Vu et al., 2018

cavourensis strain YBQ59	*cassia* Prels (Laurales: Lauaceae)	hydroxy-daidzein and 1-monolinolein	adenocarcinoma EGFR-TKI	
Streptomyces hygroscopicus strain TP-A0451	Herbaceous and arbor plants<br>Iranian plants	Pterocidin	Cancer cell<br>lines NCI-H522, OVCAR-3, SF539, and LOX-IMVI	Igarashi et al., 2006
Streptomyces laceyi strain MS53	*Ricinus communis* L. (Malpighiales: Euphorbiaceae)	Salaceyins A and B	Human breast cancer (cell line SKBR3)	Kim et al., 2006
